# Prognostic models for colorectal cancer recurrence using carcinoembryonic antigen measurements

**DOI:** 10.3389/fonc.2024.1368120

**Published:** 2024-05-30

**Authors:** Nastaran Mohammadian Rad, Odin Sosef, Jord Seegers, Laura J. E. R. Koolen, Julie J. W. A. Hoofwijk, Henry C. Woodruff, Ton A. G. M. Hoofwijk, Meindert Sosef, Philippe Lambin

**Affiliations:** ^1^ The D-Lab, Department of Precision Medicine, GROW – Research Institute for Oncology & Reproduction, Maastricht University, Maastricht, Netherlands; ^2^ Department of Surgery, Zuyderland Medisch Centrum, Sittard-Geleen, Netherlands; ^3^ St.Elisabeth Krankenhaus Geilenkirchen, Geilenkirchen, Germany; ^4^ Department of Radiology and Nuclear Medicine, GROW – Research Institute for Oncology & Reproduction, Maastricht University Medical Center, Maastricht, Netherlands

**Keywords:** colorectal cancer, machine learning, carcinoembryonic antigen, cancer recurrence, prognosis

## Abstract

**Objective:**

Colorectal cancer (CRC) is one of the most prevalent cancers worldwide. A considerable percentage of patients who undergo surgery with curative intent will experience cancer recurrence. Early identification of individuals with a higher risk of recurrence is crucial for healthcare professionals to intervene promptly and devise appropriate treatment strategies. In this study, we developed prognostic models for CRC recurrence using machine learning models on a limited number of CEA measurements.

**Method:**

A dataset of 1927 patients diagnosed with Stage I-III CRC and referred to Zuyderland Hospital for surgery between 2008 and 2016 was utilized. Machine learning models were trained using this comprehensive dataset, which included demographic details, clinicopathological factors, and serial measurements of Carcinoembryonic Antigen (CEA). In this study, the predictive performance of these models was assessed, and the key prognostic factors influencing colorectal cancer (CRC) recurrence were pinpointed

**Result:**

Among the evaluated models, the gradient boosting classifier demonstrated superior performance, achieving an Area Under the Curve (AUC) score of 0.81 and a balanced accuracy rate of 0.73. Recurrence prediction was shown to be feasible with an AUC of 0.71 when using only five post-operative CEA measurements. Furthermore, key factors influencing recurrence were identified and elucidated.

**Conclusion:**

This study shows the transformative role of machine learning in recurrence prediction for CRC, particularly by investigating the minimum number of CEA measurements required for effective recurrence prediction. This approach not only contributes to the optimization of clinical workflows but also facilitates the development of more effective, individualized treatment plans, thereby laying the groundwork for future advancements in this area. Future directions involve validating these models in larger and more diverse cohorts. Building on these efforts, our ultimate goal is to develop a risk-based follow-up strategy that can improve patient outcomes and enhance healthcare efficiency.

## 1 Introduction

Colorectal cancer (CRC) ranks as the third most prevalent cancer worldwide (1). Advances in chemotherapy and increased use of hepatic resection surgery have contributed to significant improvements in the survival rate for patients with this type of cancer (2). Despite these improvements, cancer recurrence remains a prolonged challenge, and delays in detection can compromise the effectiveness of surgical intervention (3). Studies have revealed that approximately 85% of recurrences occur within 30 months after surgery, with nearly all cases appearing within 5 years (4). Thus, it is essential to maintain continuous monitoring of patients even after successful therapeutic intervention to detect potential cancer recurrence at the earliest possible stage.

The current guidelines for identifying recurrence involve regular testing of CEA levels in post-operative patients (5, 6). CEA level is a widely used clinical marker, demonstrating associations with the occurrence and severity of CRC (7, 8). However, studies have revealed the fact that single CEA measurements lack strong prognostic potential for monitoring CRC, exhibiting a balanced accuracy of 0.65 (9, 10).

In recent years, machine learning (ML) techniques have gained significant traction in oncology (11, 12). These techniques are applied for both diagnosis and prognosis, aiming to enhance patient outcomes and optimize treatment strategies (13, 14). While ML models have been employed for recurrence prediction in CRC (see Section 2), there is a need for CRC prognostication models that simultaneously achieve high accuracy and offer clear explainability. This study aims to bridge this gap by employing ML techniques to accurately prognosticate CRC recurrence and also to identify the underlying factors contributing to it. Our contributions are three-fold:

- In this study, we apply and evaluate four various machine learning models, integrating demographic information, clinicopathological factors, and CEA measurements. Through the progressive integration of CEAs, we also investigate the minimal number of CEA measurements necessary to effectively predict recurrence.
- We use permutation importance method to identify the key clinical factors influencing our model’s predictions, providing valuable information about the variables most impactful in CRC recurrence.
- We investigate the impact of data imputation on the predictive performance of CRC recurrence models.

## 2 Related work

In recent years, CRC prognosis and diagnosis have gained attention in clinical and research areas. Commercial tools such as Oncotype DX Colon (15) and ColoPrint assay (16), which incorporate gene expression profiling, have emerged as resources for assessing the risk of recurrence. However, these tools showed a relatively modest performance (area under the receiver operating characteristic curve (AUC) of 0.63 for ColoPrint and 0.55 for OncoDefender-CRC) (14).

Previous studies on CRC prognosis applied ML through retrospective analyses on diverse data types, mainly as a single modality, including clinical, epidemiological, and genetic data (11, 12). Through the analysis of genetic data, Grudner et al. (17) predicted diverse clinical outcomes, including cancer recurrence. Their model demonstrated a stratification between recurrence and non-recurrence patients, surpassing the effectiveness of sub-categorization based on prior literature, reporting an accuracy of 0.71 for their predictions. In (11), the authors explored the feasibility of using ML models, mainly decision-tree-based learning algorithms, to predict recurrence in Stage IV CRC patients. The reported AUC score for their top-performing model was 0.76. Elsewhere, Castellanos et al. (12), employed an ensemble model to predict recurrence in Stage II-III CRC patients. Their dataset included gene expression data, protein-protein interaction details, and tumor suppressor and driver mutation information. Their experimental results showed superior predictive capabilities on molecular data compared to clinical data alone. Their most effective model achieved an AUC of 0.79. In (13), the authors applied a range of ML models on a relatively small dataset with 904 CRC patients to predict recurrence. Their best-performing model, a support vector machine (SVM), applied to the structured data yielded an AUC of 0.83.

## 3 Methods

### 3.1 Data collection

**Figure 1:**
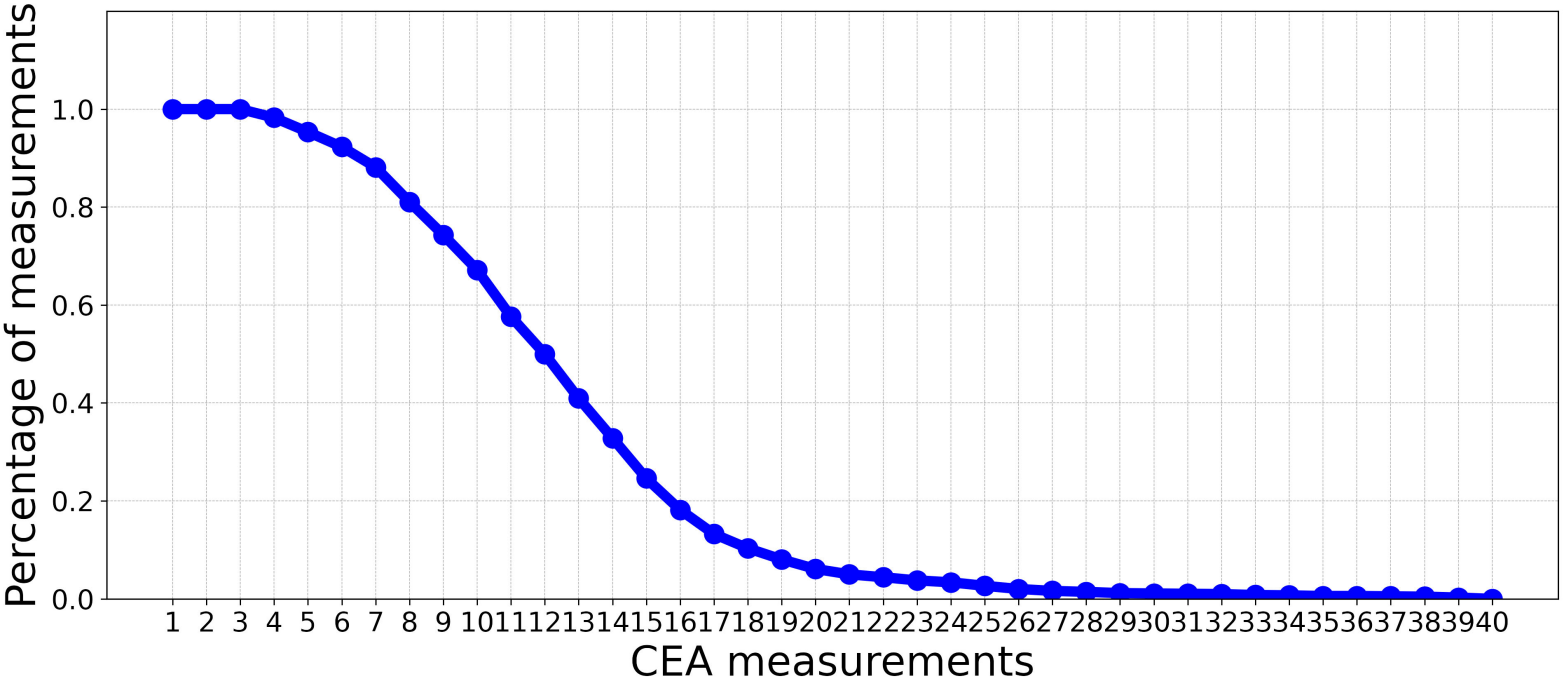
The percentage of CEA measurements (non-missing values) in each time point over all patients.

This study used a dataset of patients diagnosed with Stage I-III CRC who were referred to Zuyderland Hospital for surgery with curative intent and follow-up of the primary tumor between 2008 to 2016. This study was approved by the Medical Ethics Committee, and informed consent was not obligatory. The dataset is composed of static and time-series (dynamic) features. The static features consist of 24 predictor variables that are associated with recurrence, such as demographic information, comorbidities, tumor characteristics, and treatment parameters. As shown in **Figure 1**, the dynamic features contain 40 CEA measurements. Patients were followed up post-operatively according to the Dutch National Guidelines, every 3 to 6 months on average (for a detailed description of all included predictor variables, see **Table 1**).

**Table 1:** An overview of the different variables used for the colorectal cancer recurrence prediction model.

Category	Variable (Type)	Description
**Demographics**	Age (con) Smoking status (cat)	Age at time of surgery Divided into three classes: never, past, or current smokers
	Sex (bin)	Male or female
**Comorbidity**	Irritable Bowel Syndrome (bin) Inflammatory Bowel Disease (bin)	Gastrointestinal tract functional disorder characterized by chronic abdominal pain and altered bowel habits Chronic auto-inflammatory condition of the gastrointestinal tract
	Diabetes (bin)	Absolute or relative insulin deficiency
	Familial Adenomatous Polyposis (bin)	Presence of familial adenomatous polyposis; rare inherited disease causing extensive polyp formation
	Lynch (bin)	Presence of hereditary nonpolyposis colorectal cancer (HNPCC, or Lynch syndrome)
	Cardiac disease (bin)	Cardiac disease present (e.g., congestive heart failure, ischemic heart disease)
**Tumor Characteristics**	Organ (bin) Synchronic metastasis (bin) Location (bin) cTNM stage (cat) ycTNM (cat)	Location of tumor (colon/rectum) Presence of metastasis detected at or before diagnosis of the primary tumor Location of metastasis (liver/other location) Clinical TNM (5th edition) Clinical TNM after neoadjuvant therapy
	p(y)TNM (cat)	Pathological TNM (5th edition)
	Tumor type (cat)	Tumor type (adenocarcinoma, mucinous carcinoma, or other)
	Cancer staging (cat)	Cancer stage according to pTNM
	lymph invasion (bin)	Presence of lymph invasion
	Angioinvasion (bin)	Presence of angio invasion
**Treatment parameters**	Neoadjuvant therapy (cat)	Radiotherapy (5x5 Gray), chemotherapy, or radiochemotherapy
	Adjuvant chemotherapy (bin)	Use of any form of adjuvant chemotherapy
	Adjuvant radiotherapy (bin)	Use of any form of adjuvant radiotherapy
**Treatment outcome**	Resection marge free (bin)	Surgical outcome in achieving complete tumor removal
**Tumor marker**	CEA measurements (con)	Tumor marker used for detection recurrence

The variables are ordered based on their category. The variable types and descriptions are provided. Con, continuous; Cat, categorical; Bin, binary; TNM, TNM-classification; CEA, Carcinoembryonic antigen; HNPCC, Hereditary Non-Polyposis Colorectal Cancer; 5x5 Gray, 5x5 rectal cancer radiation protocol.

### 3.2 Data preprocessing

Comprehensive data preprocessing steps were performed to ensure the integrity of the dataset. Initially, 13 patients who presented inconsistencies in their data with the time of data collection appearing in descending order contrary to the expected chronological progression post-surgery were excluded. CEA measurements obtained before surgery were disregarded for the remaining patients. Missing data were then imputed using data imputation techniques based on each feature type. In line with previous studies (13), missing entries within each binary and categorical feature (e.g., smoking status) were imputed using the most frequent value present in that particular feature (See **Supplementary Table S1** in **Supplementary Material** for the number of missing values in each static feature). For continuous values (e.g., CEA measurements), median value-based imputation was employed, effectively maintaining the overall distribution characteristics. Then, categorical features were encoded using the one-hot encoding scheme, resulting in 67 features for subsequent modeling. Quantile transform was applied to features to mitigate the impact of outliers and non-normality in the original data. The final preprocessed dataset consisted of 1927 patients (See **Supplementary Table S2** in **Supplementary Material** for the distribution of patients by cancer stage). The dataset was imbalanced, with the positive class (recurrence) constituting approximately 15% of the total dataset which equates to 285 patients.

### 3.3 Experimental setup

#### 3.3.1 Experiment 1 (prognostic models using static features)

This experiment aims to investigate the influence of static clinical data on the accuracy of recurrence prediction in patients with CRC. We evaluated four diverse classifiers: 1) logistic regression (LR), a linear classifier; 2) support vector machine (SVM) with a radial basis function kernel, a non-linear classifier; 3) random forest (RF), a decision-tree-based classifier; and 4) gradient boosting (GB), an ensemble model of decision-tree based classifiers. Furthermore, to identify the key clinical factors contributing to the recurrence prediction, we applied the permutation importance technique (18), a model-agnostic method for assessing feature importance, on the static features using our top-performing classifier.

#### 3.3.2 Experiment 2 (prognostic models using static features and step-wise incorporation of CEA measurements)

This experiment aims to assess the impact of incorporating CEA measurements alongside static features for recurrence prediction. We evaluated the performance of the classifiers introduced in Experiment 3.3.1 using a limited number of CEA measurements after surgery. In this context, we progressively incorporated CEA measurements with static features. This iterative process involved gradually adding individual CEA measurements at a time to the existing set of static features, incrementally building a comprehensive set of combined features. Subsequent to each inclusion of a new CEA measurement, we trained ML models, outlined in Experiment 3.3, with the updated input for the prediction. As depicted in **Figure 1**, by moving beyond the first 10 CEA measurements, the percentage of measurements (non-missing values) in each time point over all the patients significantly decreases. Consequently, we have restricted our analysis to these initial 10 CEA measurements. This selection ensures a more reliable and complete dataset, with less than 50% missing values.

#### 3.3.3 Experiment 3 (the impact of data imputation)

Considering the presence of missing values in our dataset and the use of imputation as a preprocessing step, this experiment examines the impact of data imputation on recurrence prediction. This is achieved by comparing the performance of the best-performing classifier, which was applied to the imputed data, with that of the Histogram-based Gradient Boosting (HGB) classifier. Unlike all classifiers used in this study, the HGB classifier can handle missing values without data imputation. By using the HGB classifier, we aim to evaluate the impact of its missing value-handling capabilities on the predictive accuracy of our recurrence prediction task. Through a comparative analysis, we can assess the benefits of incorporating the HGB classifier’s missing value-handling mechanism in our prediction model.

All classifiers were implemented using the Sklearn library (19). To tackle the challenge of data imbalance, a weighted training approach was adopted, wherein class weights were set to be inversely proportional to their frequencies in the dataset. Hyperparameters for these classifiers were optimized using a grid search algorithm, which was applied to a validation set to ensure optimal model performance and generalizability.

### 3.4 Evaluation

In this study, the samples were randomly divided into training and testing sets at a ratio of 8:2. All the experiments were repeated 10 times to evaluate the variability in performances and ensure reliable estimates of the model’s performance. For each repetition, the following evaluation metrics were calculated to measure the classification performance:

- Area Under the Curve (AUC): This metric measures the model’s discriminative power, reflecting its ability to differentiate between the positive and negative classes accurately.
- Balanced Accuracy (BAC): This metric assesses the overall accuracy of a classification model, considering both sensitivity and specificity (20). Unlike traditional accuracy, which may be misleading in the presence of imbalanced datasets, BAC is useful when the dataset is imbalanced. BAC inherently encompasses both specificity and sensitivity, crucial metrics often employed in evaluating clinical assay performance. Therefore, in line with prevalent ML practices (13), while we prioritize models with superior AUC scores, we also value models with high BAC scores.

## 4 Results

**Table 2** shows that the LR classifier achieved the best performance of all the ML models trained on the static data, with an AUC of 0.65 and a BAC of 0.60. Furthermore, the results indicated a boost in classifiers’ performance upon adding CEA measurements. Up until the inclusion of the first 7 CEA measurements, performance enhancements were observed for GB, RF, and SVM models (AUC and BAC increased by approximately 12–17% and 7–11%, respectively). Conversely, the performance improvement of the LR model was comparatively more gradual within the same range of measurements (both AUC and BAC increased 5%). The improvement rate diminished after the inclusion of the first 7 CEA measurements.

**Table 2:** AUC and BAC measures of four ML models when trained on static data, and combination of static data and 10 CEA measurements.

Models	Experiment	BAC	AUC
LR	Static Static+10 CEA	**0.60** ± **0.00** 0.64 ± 0.00	**0.65** ± **0.00** 0.70 ± 0.00
SVM	Static Static+10 CEA	0.54 ± 0.02 0.68 ± 0.01	0.58 ± 0.02 0.74 ± 0.01
RF	Static Static+10 CEA	0.58 ± 0.01 0.71 ± 0.02	0.62 ± 0.00 0.77 ± 0.01
GB	Static Static+10 CEA	0.59 ± 0.02 **0.73** ± **0.01**	0.60 ± 0.01 **0.81** ± **0.02**

The values indicating higher performance are highlighted in bold.

**Figure 2:**
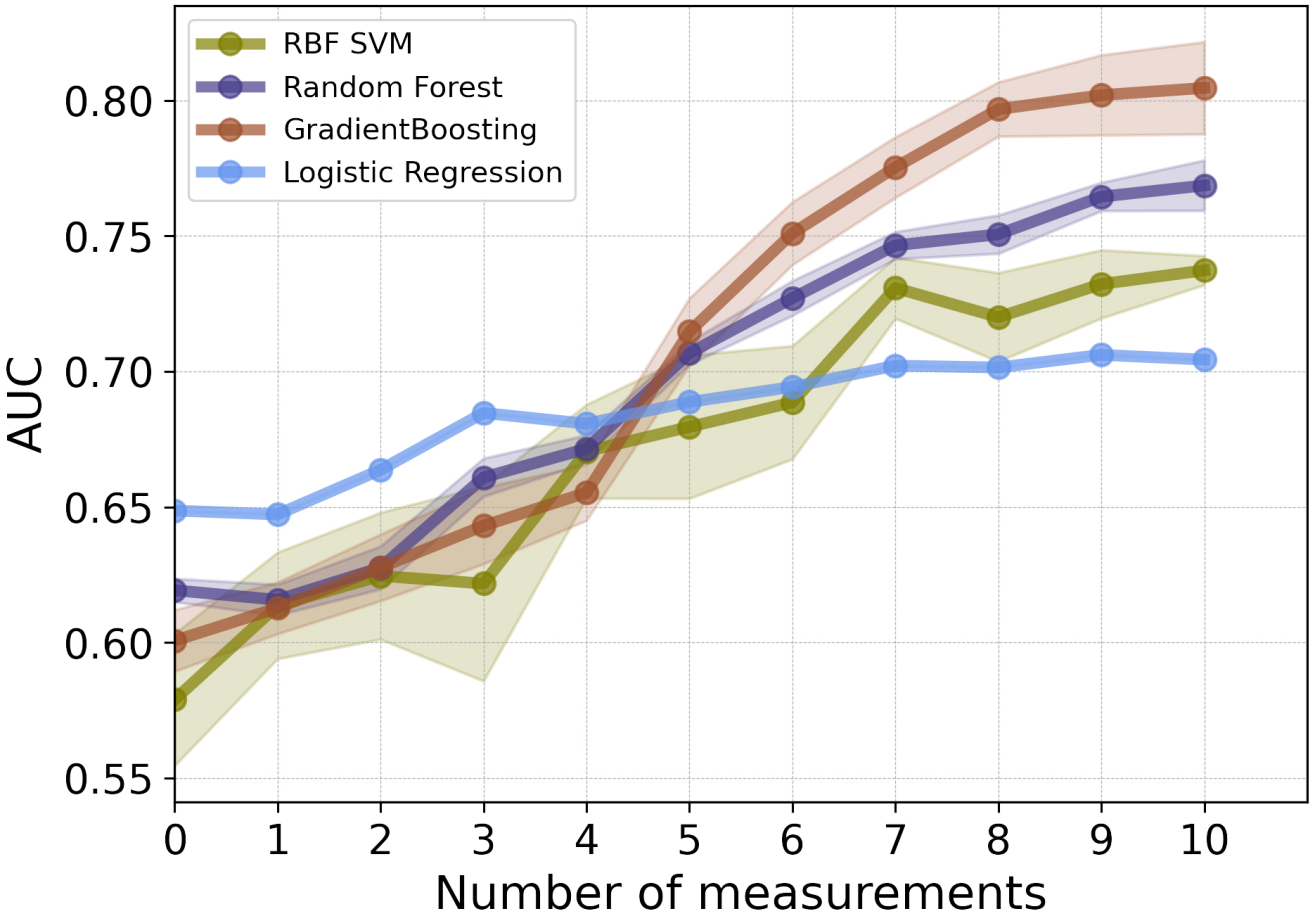
The mean AUC scores of four ML models on combination of static features and CEA measurements taken after the date of surgery.

As illustrated in **Figure 2**, for the first 3 post-operative post-operative CEA measurements, the LR model showed the highest performance in terms of AUC. Among the employed models, GB and RF classifiers outperformed other ML classifiers when applied to the combination of static and CEA measurements. GB trained on the combined static data and 10 CEA measurements performed the best, achieving the highest performance with an AUC score of 0.81 and BAC of 0.73. Furthermore, our results showed that using only the first 5 post-operative CEA measurements in combination with static data, the GB model was able to predict recurrence with an AUC of 0.71. Although this marked a reduction of around 10% from the final model’s performance, which used the entire dynamic data, the performance demonstrated an incremental enhancement with the inclusion of more time points.

**Figure 3:**
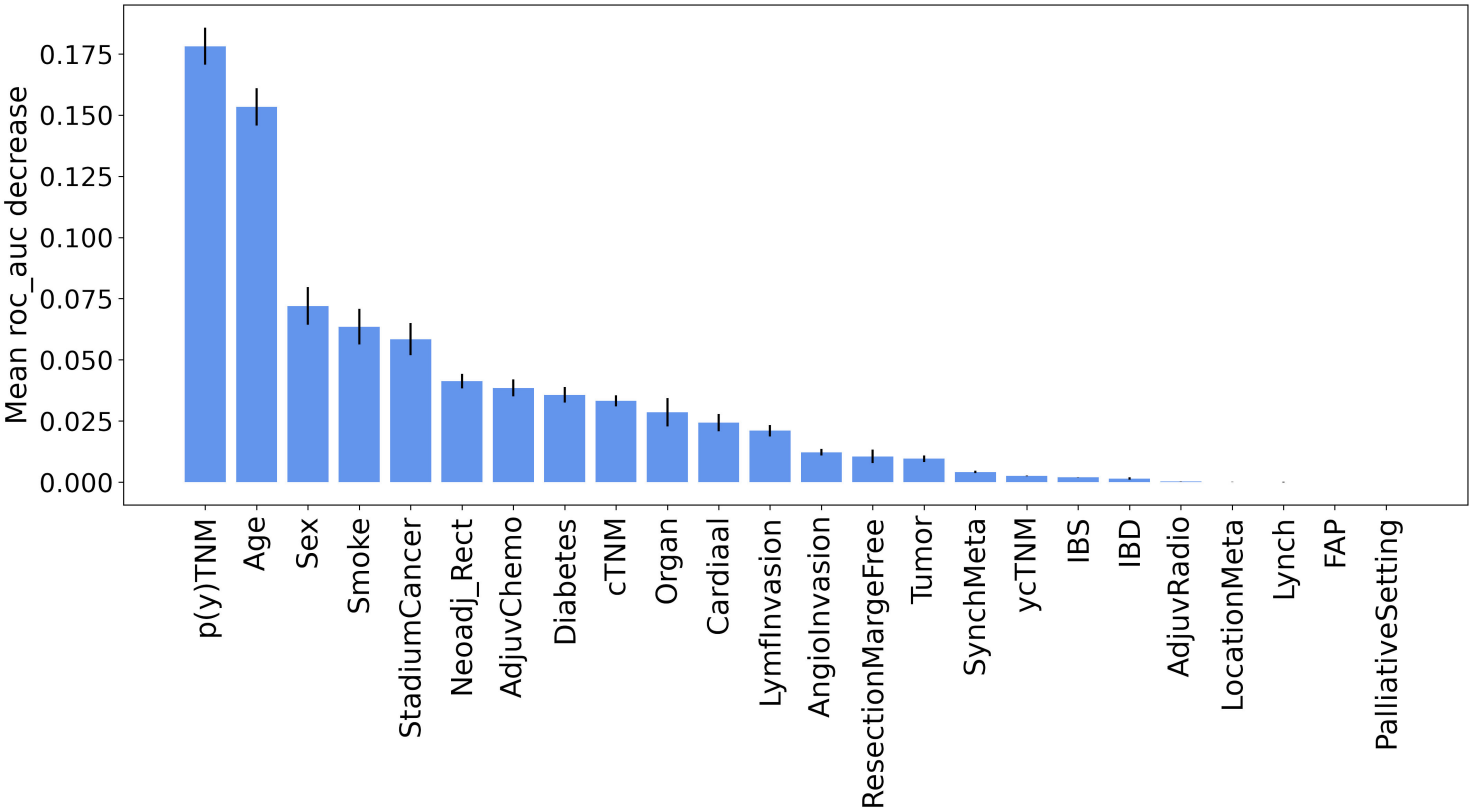
GB feature importance via permutation importance method.

Furthermore, the results of permutation importance method depicted in **Figure 3** identified tumor characteristics and demographic information as key determinants. As expected, p(y)TNM was the most important feature, demonstrating a substantial effect on the prediction of recurrence. While p(y)TNM and cancer stadium are measurements for advanced tumor stages, p(y)TNM provides a more detailed account of tumor size and metastasis. In contrast, cancer stadium is a more compressed or simplified version of the p(y)TNM classification. This simplification is primarily evident in stage III cancer, where we did not differentiate between sub-stages A, B, and C but rather considered it as a single stage. Thus, our analyses suggested that p(y)TNM remains the most detailed and informative variable for inclusion in the model, mainly because of its comprehensive detailing of the extensiveness of tumor growth and spread. Among other features, age also showed a significant influence on recurrence prediction, reinforcing its importance as a prognostic factor (21–24).

**Figure 4:**
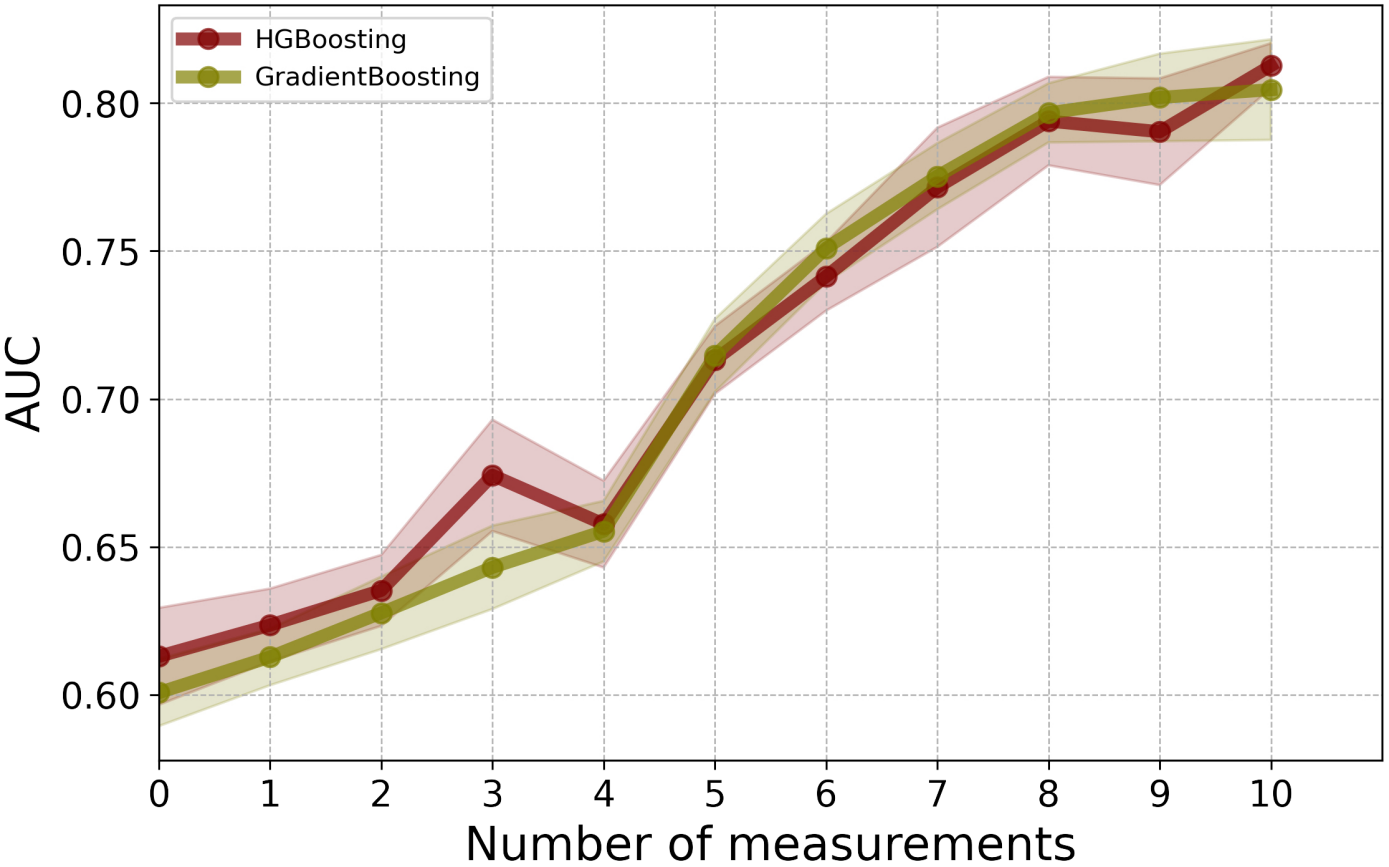
Performance comparison between GB with data imputation and HGB without data imputation.

As an alternative solution to data imputation, one can use HGB, which offers a mechanism to handle missing values directly without the need for data imputation. By comparing the results of HGB with the best-performing model, GB, which requires data imputation, we observed that HGB achieved comparable performance without the additional step of data imputation (see **Figure 4**). Using HGB can streamline the modeling pipeline and simplify the data preprocessing, ultimately leading to more efficient and reliable predictions.

## 5 Discussion

In this study, we proposed the application of ML for recurrence prediction in patients with CRC using a combination of longitudinal CEA measurements with clinical information, including demographic data, tumor staging, and treatment parameters. Our best-performing classifier, GB, achieved remarkable AUC and BAC scores. In summary, the model’s predictive ability for recurrence, based on limited and early post-surgical CEA measurements, suggests the potential for devising personalized monitoring schedules. In addition, our analysis underscored the significance of demographic information, including age and sex, as well as tumor attributes such as p(y)TNM in predicting the risk of recurrence. These findings are consistent with earlier studies, highlighting a high risk of recurrence in older patients (21–24) and align with evidence of an association between advanced tumor stages and an increased risk of recurrence (21, 25). Furthermore, the analysis suggested that the impact of comorbidities on recurrence prediction was less pronounced when compared to these other factors. Furthermore, we showed that using the HGB model can remove the need for data imputation while preserving the model’s performance.

One of the major strengths of this study is the large sample size and the availability of data on a wide range of variables. Our dataset comprises 1927 patients representing a significant increase in size compared to datasets used in prior studies (13, 14). The ample sample size in our dataset supports the application of deep learning. Considering the presence of temporal information in the longitudinal CEA measurements, recurrent neural networks are considered suitable candidates for recurrence prediction in future studies.

Despite the promising results, this study has several limitations. We evaluated our models using data collected from a single hospital while the heterogeneity of patient demographics, disease presentations, and treatment protocols across different hospitals and geographic locations can significantly influence model performance. To address this critical aspect of our research and ensure the robustness and generalizability of our models, we need to further validate the developed models on a broader range of patient data, reflecting diverse demographic and clinical characteristics. While the outcomes highlighted in this study are promising, it is worth noting that these achievements have been obtained by directly applying ML models to the raw data without involving any feature extraction processes. This shows the potential inherent in the original data to contribute to the predictive capabilities of the models. In future work, we will investigate the advantages of incorporating feature extraction methods from clinical data. This could encompass manually curating features that align with domain knowledge or deploying advanced techniques that enable the models to learn informative features automatically. Furthermore, all the analyses were conducted retrospectively. Consequently, the performance of the model in predicting cancer recurrence on new, yet-to-be-observed data could not be directly assessed or validated in real-time. The ability of the model to accurately predict cancer recurrence in future patients remains to be tested through prospective studies. By developing an application (26) to frequently capture patient symptoms in short intervals after surgery, we can bridge the gap between real-time patient experiences and medical interventions. Such a system facilitates timely prediction and management of recurrence and promotes a patient-centric approach by allowing individuals to participate in their care actively. The adoption of such platforms has the potential to revolutionize recurrence prediction and overall patient management.

Additionally, in future work, we aim to explore integrating multi-modal healthcare data, recognizing its potential to enhance the prediction of CRC recurrence. This approach will involve diverse data types, such as molecular prognostic factors (27) and incorporation of radiomic analysis (28), each contributing unique information about the disease’s progression and the prognosis of the patient. The inclusion of molecular prognostic markers, offers a deeper understanding of the tumor’s biological behavior. These markers can provide information about the aggressiveness of the cancer, its likelihood of recurrence, and potential response to therapy. Incorporating radiomic analysis into our model can enhance our understanding of the tumor’s characteristics and its interaction with surrounding tissues, further refining our predictions of recurrence risk.

## 6 Conclusion

CRC remains a significant global health challenge, with a notable percentage of patients experiencing recurrence after curative surgery. This study showed the value of CEA as a non-invasive and efficient marker for recurrence prediction. Through the application of ML, specifically GB classifier, we demonstrated an accurate recurrence prediction using comprehensive clinical data combined with CEA measurements, even when limited to early CEA measurements. We further showed that age and tumor characteristics are the most important factors influencing the risk of recurrence. Finally, we showed that HGB yields performance comparable to GB for this particular dataset while eliminating the need for data imputation. As healthcare moves towards more patient-centric models, the integration of web-based platforms and real-time symptom monitoring will be crucial. The findings of this study highlight the need for further prospective studies and show the transformative potential of ML in revolutionizing patient centered care in CRC management.

## Data availability statement

The data analyzed in this study is subject to the following licenses/restrictions: patient confidentiality and participant privacy. Requests to access these datasets should be directed to OS, odin.sosef@me.com, and JS, jordseegers@gmail.com.

## Ethics statement

The studies involving humans were approved by Medical Ethical Review Committee Zuyderland and Zuyd University of Applied Sciences (METC Z). The studies were conducted in accordance with the local legislation and institutional requirements. The human samples used in this study were acquired from primarily isolated as part of your previous study for which ethical approval was obtained. Written informed consent for participation was not required from the participants or the participants’ legal guardians/next of kin in accordance with the national legislation and institutional requirements.

## Author contributions

NM: Writing – original draft, Visualization, Methodology, Formal analysis, Data curation, Conceptualization. OS: Writing – review & editing, Resources, Data curation. JS: Writing – review & editing, Resources, Data curation. LK: Data curation, Writing – review & editing, Resources. JH: Writing – review & editing, Resources. HW: Writing – review & editing. TH: Writing – review & editing, Resources. MS: Writing – review & editing, Resources. PL: Conceptualization, Writing – review & editing, Supervision.
