# Supplementary material for: Prognostic models for colorectal cancer recurrence using carcinoembryonic antigen measurements
Source: Front Oncol. 2024 May 30;14:1368120. doi: 10.3389/fonc.2024.1368120 (PMC11169634; doi:10.3389/fonc.2024.1368120)
Supplement: Supplementary file 1 [file DataSheet_1.pdf]

Supplementary Material

1 SUPPLEMENTARY TABLES

Table S1 shows the number of missing values for each static feature and Table S2 presents the distribution of patients by cancer stage, and

1.1 Tables

Table S1. The number and percentage of missing values for static features.

Static Features	# missing values	Percentage (%)
Age	0	0
Smoking status	98	5
Sex	0	0
Irritable bowel syndrome	0	0
Irritable bowel disease	0	0
Diabetes	0	0
Familial adenomatous polyposis	0	0
Lynch	1	0.05
Cardiac disease	0	0
Organ	0	0
Synchronic metastases	4	0.2
Location of metastases	1867	97
cTNM	1345	70
ycTNM	1857	96
p(y)TNM	0	0
Tumor type	7	0.4
Cancer staging	0	0
Lymph invasion	71	3.7
Angioinvasion	71	3.7
Neoadjuvant Therapy	6	0.3
Adjuvant chemotherapy	3	0.2
Adjuvant radiotherapy	7	0.4
Resection marge free	41	2.1

Table S2. Distribution of patients by cancer stage.

Characteristic		Total number (n=1927)
Stadium cancer	Stadium I	351
	Stadium II	668
	Stadium III	849
	Stadium IV	59
